# High-throughput screening platform for solid electrolytes combining hierarchical ion-transport prediction algorithms

**DOI:** 10.1038/s41597-020-0474-y

**Published:** 2020-05-21

**Authors:** Bing He, Shuting Chi, Anjiang Ye, Penghui Mi, Liwen Zhang, Bowei Pu, Zheyi Zou, Yunbing Ran, Qian Zhao, Da Wang, Wenqing Zhang, Jingtai Zhao, Stefan Adams, Maxim Avdeev, Siqi Shi

**Affiliations:** 1 School of Computer Engineering and Science, Shanghai University, Shanghai, 200444 China; 2 State Key Laboratory of Advanced Special Steel, School of Materials Science and Engineering, Shanghai University, Shanghai, 200444 China; 3 Materials Genome Institute, Shanghai University, Shanghai, 200444 China; 4 Department of Physics and Shenzhen Institute for Quantum Science and Technology, Southern University of Science and Technology, Shenzhen, Guangdong 518055 China; 5 School of Materials Science and Engineering, Guilin University of Electronic Technology, Guilin, 541004 China; 6 Department of Materials Science and Engineering, National University of Singapore, Singapore, 117579 Singapore; 7 Australian Nuclear Science and Technology Organisation, Locked Bag 2001, Kirrawee DC, NSW, 2232 Australia; 8 School of Chemistry, The University of Sydney, Sydney, 2006 Australia

**Keywords:** Batteries, Computational methods

## Abstract

The combination of a materials database with high-throughput ion-transport calculations is an effective approach to screen for promising solid electrolytes. However, automating the complicated preprocessing involved in currently widely used ion-transport characterization algorithms, such as the first-principles nudged elastic band (FP-NEB) method, remains challenging. Here, we report on high-throughput screening platform for solid electrolytes (SPSE) that integrates a materials database with hierarchical ion-transport calculations realized by implementing empirical algorithms to assist in FP-NEB completing automatic calculation. We first preliminarily screen candidates and determine the approximate ion-transport paths using empirical both geometric analysis and the bond valence site energy method. A chain of images are then automatically generated along these paths for accurate FP-NEB calculation. In addition, an open web interface is actualized to enable access to the SPSE database, thereby facilitating machine learning. This interactive platform provides a workflow toward high-throughput screening for future discovery and design of promising solid electrolytes and the SPSE database is based on the FAIR principles for the benefit of the broad research community.

## Introduction

Historically, new materials developments have conventionally been driven by a trial-and-error experimental approach. However, the recently established Materials Genome Initiative (MGI^1^) has provided an alternative route that can effectively reduce the development time for new materials. The critical idea behind the MGI is the combination of high-throughput computations, high-throughput experiments, and materials databases^1^. Over the past decade, many high-throughput computational materials databases have emerged, including Materials Project^2^, AFLOW^3–5^, OQMD^6,7^, NOMAD^8^, NIMS^9^, NIST^10^, AiiDA^11^ and so on. These databases contain a broad range of crystal structure and computationally derived property data, such as the formation energy, band gap, band structure, elastic constants, etc. However, they rarely include the ion-transport properties of solid electrolytes, which are crucial for research on all-solid-state batteries that are evaluated on their safety, stability, and cycle life^12^. The ion transport usually involves ion hopping from one interstitial site to another interstitial site or to a vacant lattice site with sufficiently low migration barrier energy. Factors such as the crystal structure, size of mobile ions, bottleneck size, and bonding characteristic determine this barrier energy^13^. Currently, widely used methods for calculating the ion-transport barrier include classical or *ab initio* molecular dynamics^14,15^, kinetic Monte Carlo, and nudged elastic band (NEB^16^) method, of which the NEB is an effective algorithm for the calculation of transition-state energies.

To accelerate the development of all-solid-state batteries with high energy and power densities, the high-throughput automated screening of solid electrolytes with excellent ion-transport performance is essential^17–19^. However, the automated process is limited by the complicated manual preprocessing currently required for accurate ion-transport algorithms such as the first-principles nudged elastic band (FP-NEB) method. For example, the atomate tool^20^ developed by Materials Project implements an automatic workflow for NEB calculation; however, the endpoints of the migration path for each structure must still be defined manually. In this context, we develop a high-throughput screening platform for solid electrolytes (SPSE: https://www.bmaterials.cn), that provides the following three main advances:

1. Geometric analysis^21,22^ is combined with the bond valence site energy method^23^ to rapidly simulate the path and energy profile of ion migration, facilitating the completion of high-throughput automated calculations using the FP-NEB method without requiring complicated manual preprocessing.
2. High-throughput hierarchical screening for solid electrolytes is achieved by using extremely fast empirical methods to identify promising candidates^24^ for further *ab initio* calculations, thereby accelerating the discovery of optimal solid electrolytes.
3. A materials database containing ion-transport properties is built that allows users to explore the properties of solid electrolytes. The computational data available in the materials database can be also used in machine-learning algorithms to predict and optimize materials properties.

## Results

### Platform architecture

**Fig. 1:**
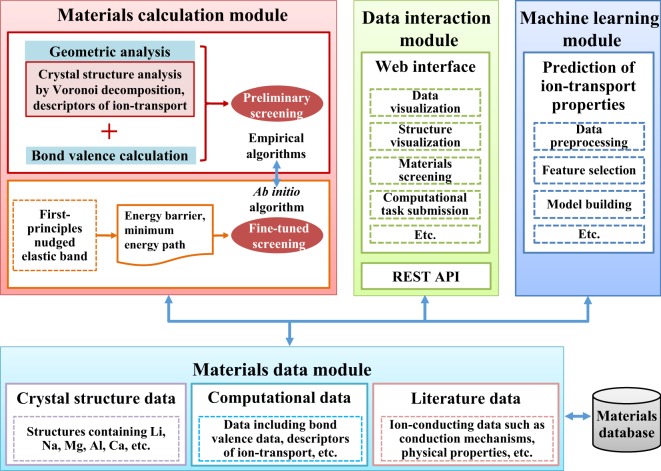
Architecture of high-throughput screening platform for solid electrolytes. The blue bidirectional lines indicate dataflow.

The objective of the SPSE platform is to provide insight into ion-transport properties to enable the materials community to explore promising solid electrolytes. To accelerate materials discovery, we design the platform architecture to include four modules: Materials data, Materials calculation, Data interaction, and Machine learning (Fig. 1). Here, we introduce the four modules of SPSE, which interact with each other.

The materials data module contains crystal structure data, computational data, and literature data. All the data are stored in a database using a MongoDB backend^25^, which is a NoSQL database based on distributed file storage. The BSON format supported by MongoDB allows the flexible storage of diversified materials data.

The algorithms of the materials calculation module can be mainly classified into two categories: empirical algorithms (geometric analysis and bond valence site energy calculations) and *ab initio* algorithm (such as first-principles nudged elastic band), which are used for preliminary and fine-tuned screenings of materials, respectively. Promising solid electrolytes can be identified and ranked using this hierarchical screening process.

One of the important components of SPSE is the data interaction module used to access our database. The data interaction module is realized in the form of a web interface implemented in the Django web framework^26^ and RESTful API^27^, which provides data access via the Hypertext Transfer Protocol (HTTP).

Finally, the machine learning module can accelerate the prediction of materials properties by extracting knowledge from data in our database to build models.

### Materials data: materials database

#### Data composition

The SPSE database implements the FAIR data principles, which ensures the processed and produced data will be findable, accessible, interoperable, and reusable^28^. As mentioned earlier, there are three main types of data in the SPSE: crystal structure, computational, and literature data, which are related to each other via independent identifier, making the data *findable*. The data can also be retrieved using a web interface, rendering it *accessible*. Moreover, the ability to download the data from the web interface reflects its *interoperability*. Finally, to ensure the *reusability* of data, the computed data retain metadata attributes (such as the calculation conditions and methods).

Currently, our database contains 91,763 crystal structures, more than 10,000 computationally derived properties (ion-transport data), and 121 properties obtained from literature for Li- and Na-containing compounds. The crystal structure data are mostly extracted from the Inorganic Crystal Structure Database (ICSD^29^) and complemented by recent literature data. The structures from the ICSD include 91,688 Li-, Na-, Mg-, Al-, Ca-, Cu-, Ag- and Zn-containing compounds. In addition, we generate 75 custom crystallographic information files (CIFs) from crystal structures data obtained from literature, with the file format of custom CIF mainly following that of the ICSD^30^. We also obtain preliminary ion-transport data for 7,678 structures through geometric analysis and 12,000 activation energy values through bond valence site energy calculations. The literature data can be roughly classified into structural information, descriptors of dynamics, conduction mechanisms, and physical properties.

#### Data storage

To ensure high efficiency of a data query, the materials data are stored separately in different collections of MongoDB. A collection is analogous to a table in a relational database management system and can store an infinite number of documents. A record is stored as a document in MongoDB; however, large data are stored in GridFS collections because of the document size limit of 16 MB.

The Crystallographic Information File (CIF) format is commonly used for storing crystal structure data; the structural information can be extracted using pymatgen^31^ or Atomic Simulation Environment (ASE)^32^. Here, the CIF data are stored in a collection after being extracted using ASE. The computational data are automatically stored in separate collections according to the calculation type.

### Materials calculation: ion-transport calculations

**Fig. 2:**
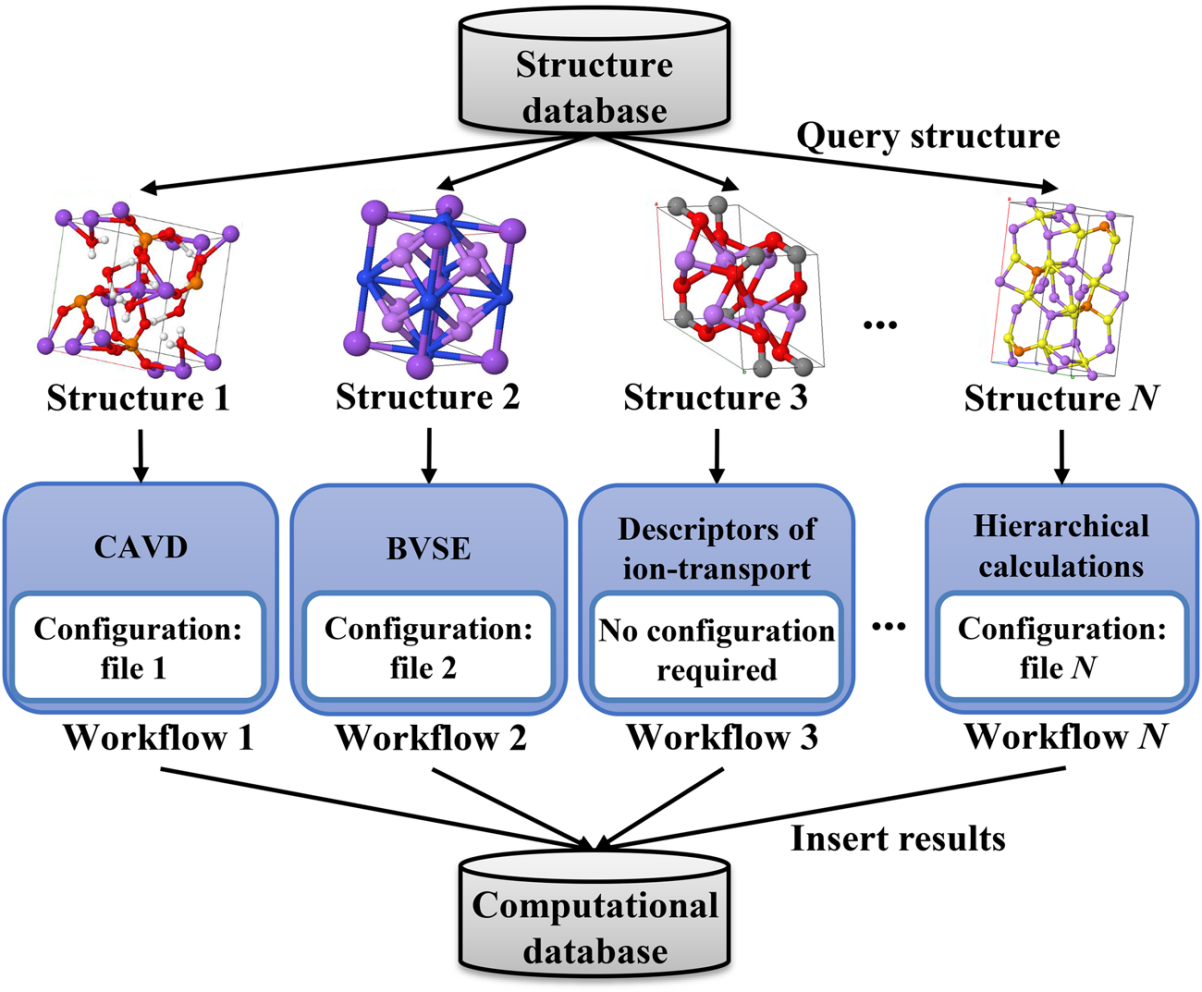
Workflow of *N* computational tasks for high-throughput automated calculations.

To enhance the computational throughput, our platform is designed to maximize its computational efficiency. The workflow of fully automated calculations is illustrated in Fig. 2. There are *N* tasks simultaneously running in the computational queue. The running of multiple concurrent jobs is managed using FireWorks^33^ and the SLURM^34^ job scheduling system. For each task, the structure is first retrieved from the database, and the configuration file is then read to execute the computational task. These computational tasks include crystal structure analysis by Voronoi decomposition (CAVD), bond valence site energy (BVSE), ion-transport descriptor, and hierarchical (i.e., CAVD + BVSE→NEB) calculations. Next, we discuss the ideas behind the CAVD, BVSE, and hierarchical calculations.

#### Crystal structure analysis by Voronoi decomposition

**Fig. 3:**
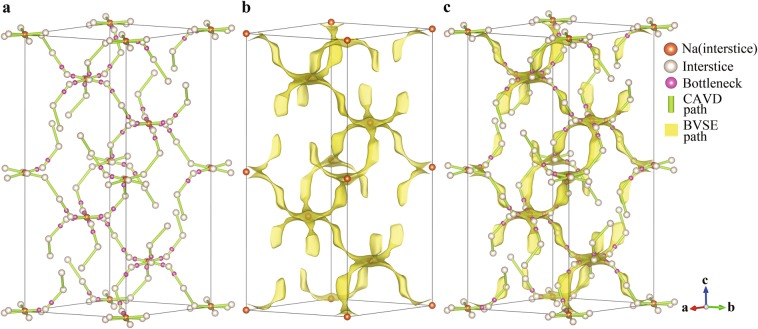
Three-dimensional migration paths of NaZr_2_P_3_O_12_ constructed using (**a**) CAVD and (**b**) BVSE calculations with migration energy barrier *E*_a_ of 1.055 eV. (**c**) Comparison of migration paths of NaZr_2_P_3_O_12_ constructed using BVSE and CAVD calculations.

The crystal space can be divided into two non-intersecting topological subspaces: the subspace of atoms and the subspace of interatomic interstices^21^. To characterize and analyze these two subspaces, we develop the crystal structural geometric analysis program CAVD^35^. In the CAVD calculation process, the interstitial network is first obtained from the subspace of atoms in the crystal structure by radical Voronoi decomposition^22^. The interstitial network consists of interstices (vertices), passageways between interstices (edges), and bottlenecks (the smallest cross-sectional areas of the passageways). The ion-transport network (also represents ion migration paths) can then be constructed by comparing the radii of mobile ions with that of interstices and bottlenecks in the interstitial network. Analysis of the interstitial network also provides the radii of the largest free sphere that can travel within the structure^22^. Similar analysis is implemented in PLATON^36^, ToposPro^37^ and Zeo++^22^ programs, but they are not suitable for automated unsupervised workflows. An example of an ion-transport network calculated using CAVD is presented in Fig. 3a for NaZr_2_P_3_O_12_^38,39^ (ICSD-467), a prototype composition from which Na superionic conductor (NASICON) solid electrolytes can be derived by ionic substitutions^40^. The threshold parameter related to the radius of a mobile ion (Na^+^) is determined to be 0.9 Å. Although the CAVD program can determine the ion-transport network of a crystal structure within seconds, we want to further characterize the network with the migration energy barrier, which is calculated using the bond valence site energy method.

#### Bond valence site energy calculation

The bond valence (BV) theory is derived from Linus Pauling’s principle of electrostatic valence^41^ and has evolved into a method for predicting the structure and bonding geometry of complex materials^23^. Currently, the BV method is used to predict ion migration paths and energy barriers^42–44^. Based on the BV method, the bond valence site energy (BVSE)^45,46^ model was developed by Adams and Rao, and bond valence energy landscape (BVEL) was proposed by Sale and Avdeev^47^. The difference between these two closely related empirical methods is, besides technical details in the pathfinding algorithm, in nuances of consideration for Coulomb repulsion. In this work, we develop a separate BVSE calculation program based on the BVSE model, which can be used to conduct the migration pathway and barriers calculations for mobile ions including Li^+^, Na^+^, Mg^2+^, Zn^2+^, Al^3+^, F^−^ etc. and is subject only to the limitations of the bond valence site energy method itself^23^.

The BVSE for a mobile ion *M* at a given site in the crystal structure is related to the sum of a Morse-type potential term for cation-anion pairs (representing both the attractive ionic, covalent or Van der Waals interactions and the Born repulsion) and Coulomb repulsions between the mobile ion *M* and the *N* immobile ions *M*_*i*_ as follows:

1 $$BVSE(M)=\frac{{D}_{0}}{2}\left\{{\left(\exp \left[\alpha \left({R}_{\min }-R\right)\right]-1\right)}^{2}-1\right\}+\mathop{\sum }\limits_{i=1}^{N}{E}_{{\rm{Coulomb}}}(M-{M}_{i}).$$

The Morse-type potential is characterized by the empirical BV parameters: *D*_0_, *α*, and *R*_min_^48,49^. The use of *D*_0_/2 as the Morse bond breaking energy prevents double-counting of the same interaction in both the energy landscapes of *M* and *M*_i_ and may be seen as taking into account in a simplified average way relaxations in the immobile substructure, as the suppression of relaxations in the static BVSE modelling tends to overestimate the migration barriers. The Coulomb repulsions between two different cations (or between anions) *M*_1_ and *M*_2_ is calculated by the following formula:

2 $${E}_{{\rm{Coulomb}}}({M}_{1}-{M}_{2})=\frac{{q}_{{M}_{1}}{q}_{{M}_{{\rm{2}}}}}{{R}_{{M}_{1}-{M}_{2}}}erfc\left(\frac{{R}_{{M}_{1}-{M}_{2}}}{{\rho }_{{M}_{1}-{M}_{2}}}\right),$$

where *q* refers to effective charge of atom, and ${R}_{{M}_{1}-{M}_{2}}$ is the distance between *M*_1_ and *M*_2_. The screening factor *ρ*_*M*1__ − *M*2_ = 0.74 × (*r*_*M*1_ + *r*_*M*2_), therein ${r}_{{M}_{i}}$ is modelled in analogy to the real part of the Ewald summation ensuring that the repulsive Coulomb interactions converge over a similar length scale as the attractive interactions. Here we use a fixed scaling factor 0.74 for the radii sum of the interacting ions in the screening factor. It may be noted that the screened Coulomb term in Eq. (1) is in contrast to the Morse term not divided by 2, which is empirically found to strengthen the relative influence of the short range Coulomb repulsion between adjacent mobile and immobile cations (or mobile and immobile anions), which helps to eliminate unphysical paths, while it allows to keep the screening factor small enhancing the computational efficiency. As shown in our recent work, (see e.g.^50^ and references therein) the chosen approach yields a semiquantitative agreement of migration barriers with the available DFT or experimental information. Using this formulation revised with respect to the original^46^ will ensure consistency of the results of SPSE and the current version of softBV^51^.

Adams developed the softBV^49,51^ software to calculate ion migration energy and profiles based on this BVSE approach, but softBV does not provide an application programming interface that can be easily integrated into the SPSE. Moreover, the aim of softBV is rather to substitute *ab initio* calculations, while in the present software suite the BVSE calculations are a step to automatically guide the first principles calculations. Hence softBV compromises to some extent on robustness, computational efficiency and transferability in order to enhance precision of the predicted energy landscape, whereas for the screening application a fast and robust approximate estimate of the migration barriers is aimed for and the precise barriers will be derived at the subsequent first principles stage.

We checked for a wide range of Li^+^, Mg^2+^, Ag^+^ compounds that the standalone softBV programme and the current BVSE programme yield closely similar results, though the algorithms differ slightly to optimize the compromise between computational efficiency, robustness and transferability for the respective application. The main difference in the calculation of the energy landscape is that the current software uses a universally fixed scaling factor 0.74 for the radii sum of the interacting ions in the screening factor ${\rho }_{{M}_{1}-{M}_{2}}$, whereas the softBV software iteratively adapts the screening factor based on the balance between Morse and Coulomb interactions in the individual structure. While the iterative approach yields higher precision results when applied to reliable fully ordered crystal structure models, it is slower, requires knowledge of bond valence parameters for all atom pairs in the crystal structure (whereas for the present algorithm knowledge of the bond valence parameters for interactions between the mobile ion and its counterions is sufficient) and the adjustment is more susceptible to be systematically biased towards too low barriers for low quality crystal structures with implausible interatomic distances. Thus for the prescreening stage of crystal structures in this work, the fixed scaling factor 0.74 is considered to be more robust.

Another difference is that softBV analyses migration barriers between local minima of the energy landscape irrespective of their site occupancy leading to a focus on comprehensively mapping interstitial sites, while in this work the BVSE approach is primarily meant to guide the first principles calculation of energy barriers between the occupied sites in the crystal structure reducing the need to explicitly classify and analyze interstitial sites. The BVSE calculation program generates a periodic grid volumetric data (GRD) file^52^ for visualization of the ion migration paths. An example of the three-dimensional migration paths visualization is presented in Fig. 3b for NaZr_2_P_3_O_12_.

#### Hierarchical calculations

**Fig. 4:**
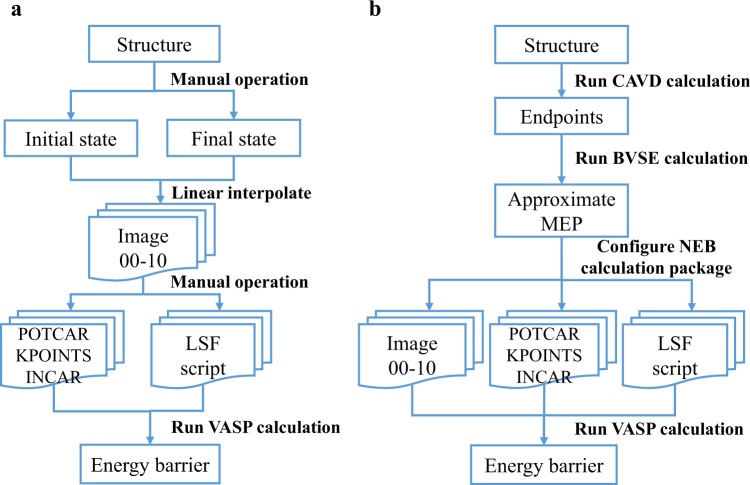
Comparison of calculation processes between (**a)** NEB and (**b)** CAVD + BVSE→NEB. Two manual operations are required to generate the initial and final states and configuration files for VASP calculation in (**a)**, while these configurations in (**b)** are automatically completed.

The NEB is an efficient approach for finding the minimum energy path (MEP) between the given initial and final states of a transition^16,53,54^, but requires complicated data preprocessing before NEB calculation can be done, for example, with the Vienna *Ab Initio* Simulation Package (VASP)^55,56^. The preprocessing includes locating the initial and final states of ion migration, configuring input files, and generating a set of transition states (images) by linear interpolation (Fig. 4a). Moreover, the MEP constructed by linear interpolation may have an image with an unphysical distance between atoms^57^. In this process, human intervention is unavoidable. To overcome these issues, we develop high-throughput automated hierarchical algorithms that combine empirical CAVD and BVSE calculations to identify the approximate MEPs of ion migration, avoiding unphysical paths. A more accurate migration energy barrier can be further obtained by fine-tuning the observed MEP using FP-NEB calculation (Fig. 4b).

The NEB calculation requires simultaneous optimization of a set of transition states. The iterative optimization is performed until the NEB force and energy criteria are satisfied to obtain the MEP. The NEB force contains two independent components,

3 $${F}^{{\rm{NEB}}}={F}^{{\rm{T}}}+{F}^{{\rm{S}}},$$

where *F*^T^ is the component of true force due to the potential perpendicular to the band and *F*^S^ is the spring force parallel to the band^58^. During the optimization process, the images are affected by the spring forces. To ensure that an equal spacing between the images is maintained along the path, the spring forces must be updated at each iteration. An important feature of the hierarchical algorithm is that the calculation of the migration path is performed using the simplified string method^59^, which in contrast to the NEB method does not require definition of the spring force along the path or the use of force projections, eliminating instability issues associated with the NEB method^58^ that are particularly detrimental for the intended automated pathway analysis. Rong *et al*. also used the simplified string method to accelerate the construction of the MEP^57^; however, they use the density functional theory-derived scalar charge density as the basis of true force definition, whereas we utilize the computationally efficient empirical BVSE approach. The standardized hierarchical flow of the calculations can be therefore summarized as follows (only works in this way for fully ordered structures, which without sites with mixed or fractional occupancies):

(1) The use of CAVD + BVSE to find the endpoints of ion migration paths

To enable automatic calculations, we use CAVD + BVSE to find the endpoints of the ion migration path in an ordered structure. The ion-transport network calculated using CAVD is mapped to an undirected graph *G* (*V*, *E*), which consists of a set of vertices (*V*, including interstices and bottlenecks) and edges (*E*) that connect a pair of vertices; BVSE values are used to characterize these vertices and exclude vertices of high energies to obtain more reliable ion-transport network *G'* (*V*, *E, B*), of which *B* represents that BVSE value of each vertex. Generally, the mobile ions at lattice site locations are characterized by relatively low site energies. Thus, we choose adjacent lattice sites as endpoints of the migration path and use *S* to denote a set of lattices sites. As adjacent lattices sites are not necessarily connected, we use the ion-transport network *G*′ to screen out connected path segments for adjacent lattices sites. Considering the efficiency of the algorithms, we construct the non-equivalent path segments by excluding duplicate equivalent path segments. The criterion for judging the equivalent path segments is equivalent endpoints and equivalent interstices, which are the components of the path. The concept of equivalent path (endpoints, interstices) is similar to that of equivalent atoms. In other words, one path segment can be used to generate a set of equivalent path segments via symmetry operations. The non-equivalent path segments are then used to locate endpoints of migration paths: $P=\{(x,y),x\in S,y\in S,x\ne y\}$.

(2) The use of BVSE calculation to determine approximate MEP

BVSE calculations yield a three-dimensional mesh composed of energy values of grid points in a unit cell, with a default distance between two adjacent grid sites of 0.1 Å. A mobile ion in the three-dimensional grid tends to move toward the adjacent grid site of minimal BVSE value (i.e., the energetically stable site), avoiding unphysical distances with other atoms. Consequently, the BVSE energy landscape can be used to simulate the potential force field to calculate *F*^T^ in Eq. (3). This information is combined with the simplified string method to calculate the approximate MEP between each endpoints (*x*, *y*) in *P* (this process takes an average of 5 min for one structure).

(3) Configuration of NEB calculation package

When using the approximate MEP determined by CAVD + BVSE calculations as the initial path for the NEB calculation, the intermediate images will be produced along the approximate MEP by interpolating between the initial and final structures. The initial and final structures are created by removing one atom from the endpoints, and other input files (INCAR, POTCAR, and KPOINTS) are automatically generated using pymatgen. In addition, we define a template for Load Sharing Facility (LSF) script. All the files are packaged as the NEB calculation package which can be directly employed to run the VASP calculation, and manual preprocessing is no longer needed.

**Fig. 5:**
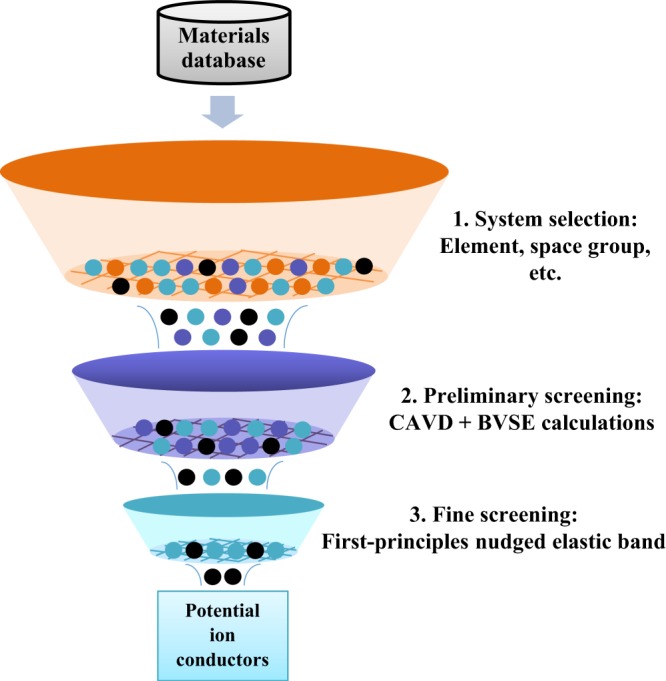
Screening process for solid electrolytes based on hierarchical ion-transport algorithms.

The hierarchical algorithms can be applied for materials screening (Fig. 5). First, candidates are selected from the SPSE database by imposing certain arbitrary conditions, e.g. on composition. Second, high-throughput preliminary screening of materials is performed using empirical algorithms (such as CAVD and BVSE calculations). Finally, *ab initio* algorithm (such as first-principles nudged elastic band) is used for fine-tuned screening of materials to identify potential solid electrolytes. The complete example of hierarchical algorithms in the SPSE is as follows:

Step 1. Preliminary results

There are 21,542 candidate compounds containing Li and Na in SPSE, of which only 5,192 candidates remained after the preliminary screening for *E*_a_ ≤ 1.2 eV in one-dimensional migration paths (see Supplementary Information S1). These 5,192 compounds include NaZr_2_P_3_O_12_ and Li_7_La_3_Zr_2_O_12_ (LLZO, ICSD-246817). The garnet-related LLZO is a lithium conductor with a high-conductivity cubic phase and low-conductivity tetragonal phase^60,61^. The thermodynamically stable phase of LLZO at room temperature is the tetragonal phase^62,63^. NaZr_2_P_3_O_12_ with space group *R-*3*c* (no. 167) and tetragonal LLZO with space group *I*4_1_*/acd* (no. 142) are used below as examples of the hierarchical calculations.

Step 2. Finding endpoints of ion migration paths

**Fig. 6:**
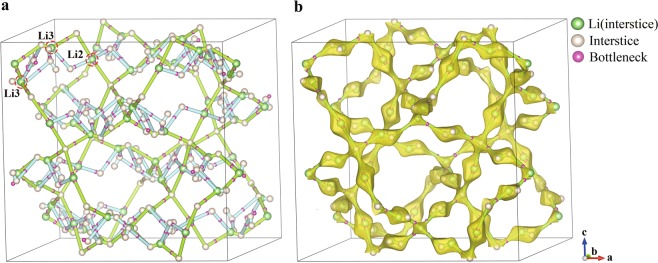
(**a**) Three-dimensional migration paths of tetragonal LLZO generated using CAVD are shown in green and blue cylinders, where the threshold parameter related to the Li^+^ radius is 0.563 Å. (**b**) Comparison of migration paths of tetragonal LLZO calculated using CAVD and BVSE. The 3D migration paths of BVSE are shown in yellow isosurfaces with migration energy barrier *E*_a_ of 0.576 eV.

The consistency of the CAVD and BVSE calculation results is an important premise for the hierarchical calculations and is verified by visualizing the migration paths of NaZr_2_P_3_O_12_ and tetragonal LLZO (Figs. 3 and 6). The visualization demonstrates that the interstices and bottlenecks appear at the minimum and maximum energy sites of BVSE, respectively; the three-dimensional migration paths calculated using CAVD are in excellent agreement with the BVSE results (Figs. 3c and 6b).

**Fig. 7:**
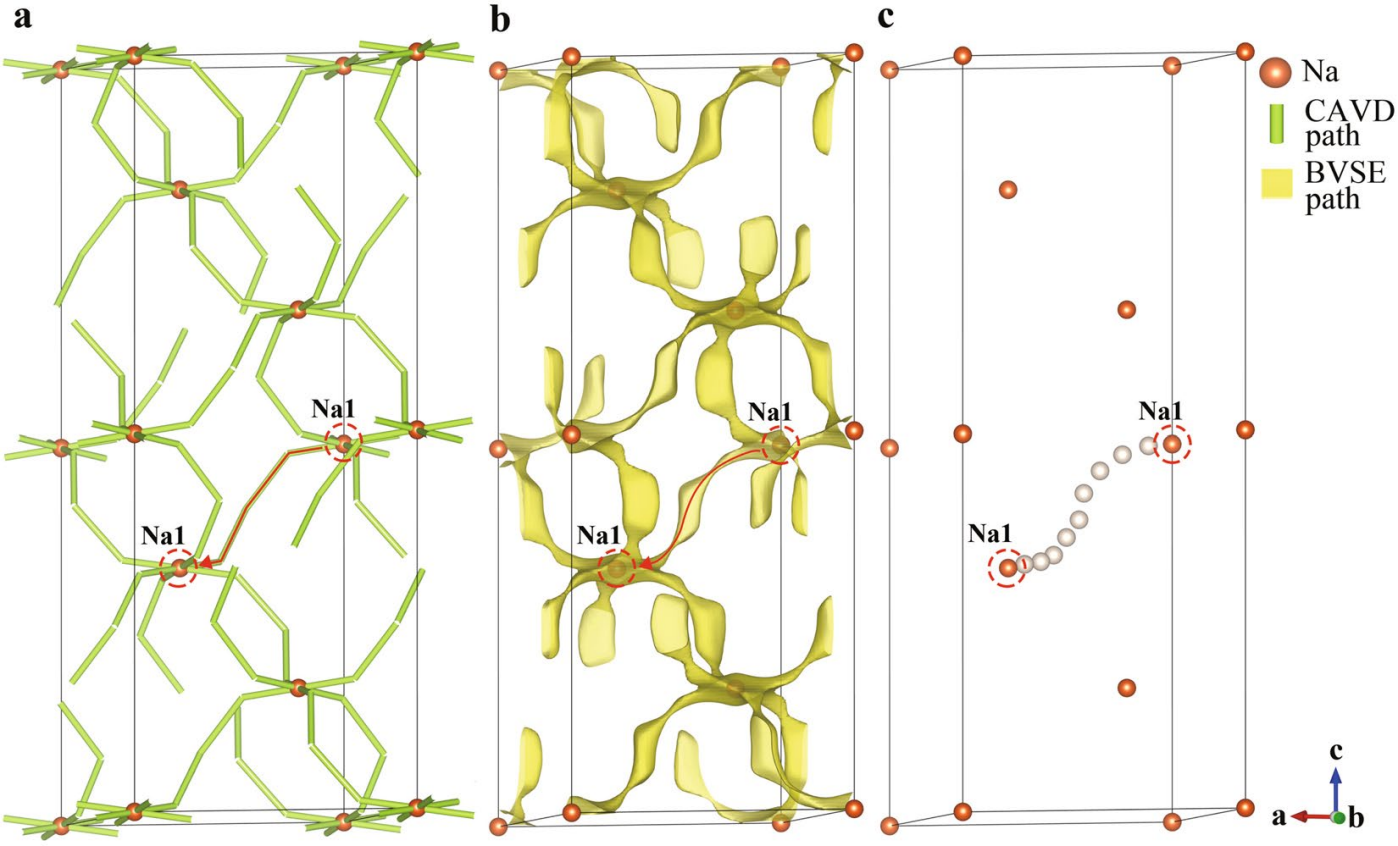
Ion migration paths of NaZr_2_P_3_O_12_ calculated using (**a**) CAVD and (**b**) BVSE respectively, where *E*_a_ is 1.055 eV. (**c**) Approximate MEP of Na1–Na1. The dotted circles indicate the endpoints.

**Fig. 8:**
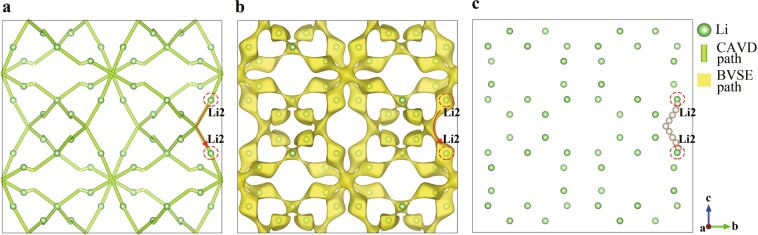
Ion migration paths of tetragonal LLZO calculated using (**a**) CAVD and (**b**) BVSE respectively, where *E*_a_ is 0.576 eV. (**c**) Approximate MEP of Li2–Li2. The dotted circles indicate the endpoints.

NaZr_2_P_3_O_12_ consists of a three-dimensional network of tetrahedral PO_4_ corner-sharing with octahedral ZrO_6_, with the Na^+^ occupying the octahedral 6b (Na1) sites^39^. A single identified non-equivalent path segment is formed by adjacent Na1 in the ion-transport network, based on the CAVD + BVSE calculations (Fig. 7a). For the tetragonal LLZO, Li atoms occupy three types of crystallographic sites: the tetrahedral 8a (Li1) sites, the octahedral 16f (Li2) sites, and the 32 g (Li3) sites^62,63^. Six types of migration paths may be considered between lattices sites: Li1–Li1, Li1–Li2, Li1–Li3, Li2–Li2, Li2–Li3, and Li3–Li3. There are two paths between Li2–Li3 and Li3–Li3 in the ion-transport network calculated by CAVD (Fig. 6a). According to the BVSE calculation, the energy barriers of the blue paths are higher than that of the green paths; therefore, the blue paths are removed to obtain a more reliable ion-transport network (Fig. 6b). Li1–Li1 and Li1–Li2 are observed to be connected via Li3 in the ion-transport network, indicating that the Li1–Li3–Li3–Li1 path can be described as concatenation of two path segments of Li1–Li3. Thus, only the other four types of non-equivalent path segments remain (one path segment is shown in Fig. 8a; further details are provided in Supplementary Information S2).

Step 3. MEP calculation

**Fig. 9:**
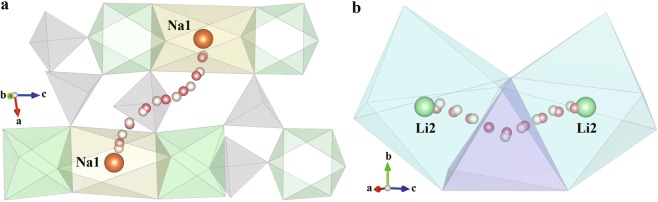
Migration paths in (**a**) NaZr_2_P_3_O_12_ and (**b**) tetragonal LLZO. The paths calculated using NEB and CAVD + BVSE are colored in pink and white, respectively. The tetrahedral PO_4_ and octahedral ZrO_6_ sites are colored gray and green, respectively. The octahedral Na1 and Li2 sites are shown in yellow and blue polyhedra respectively, and tetrahedral Li3 sites in LLZO are shown in purple.

**Fig. 10:**
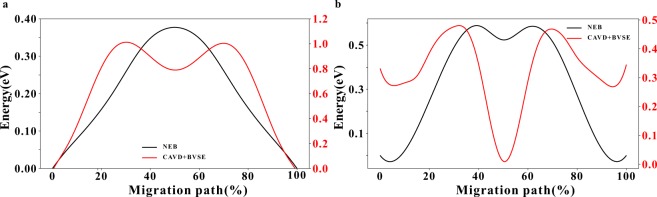
Migration energy profiles of (**a**) Na1–Na1 in NaZr_2_P_3_O_12_ and (**b**) Li2–Li2 in tetragonal LLZO calculated using NEB and CAVD + BVSE.

BVSE is used to calculate the migration paths of NaZr_2_P_3_O_12_ (Fig. 7b) and tetragonal LLZO (Fig. 8b) to determine the approximate MEP between the endpoints (Figs. 7c and 8c, respectively). The approximate MEP is consistent with the path calculated by CAVD. To evaluate the reliability of the approximate MEP, we compare the MEPs calculated using our method with those calculated using the NEB method (see Table 1 for configure parameters) and observe that the paths are fully consistent (Fig. 9). The results of Fig. 10 indicate that these two migration paths pass through two bottlenecks and one interstice and the difference in the energy profile shape is the result of the static nature of the BVSE calculations in contrast to NEB which allows local structure relaxation. In addition, the Coulomb repulsion between mobile ions is not considered in BVSE; therefore, the energy values near the bottlenecks are lower. For example, we tested one migration path of *β*-Li_3_PS_4_^64^, where the Coulomb repulsion between mobile ions was eliminated (see Supplementary Information S3).

**Table 1:** Parameters of NEB calculations.

Identifier	Compound	Space group	a (Å)	b (Å)	c (Å)	K-points set	Cut-off energy (eV)
ICSD_000467	NaZr_2_P_3_O_12_	R-3c	8.815	8.815	22.746	1 × 1 × 1	520
ICSD_246817	Li_7_La_3_Zr_2_O_12_	I4_1_/acd	13.1279	13.1279	12.6715	2 × 2 × 2	600

Overall, the comparison of the BVSE + CAVD and NEB results clearly demonstrates that the empirical methods can effectively identify the robust path of ion migration for further *ab initio* calculations.

Step 4. NEB calculation package

After determining the approximate MEP of ion migration, POSCAR files corresponding to the images along the MEP can be generated. To facilitate the VASP calculation, each POSCAR file is stored separately in folders labeled “00”-“10” (for example, nine intermediate images are generated). In addition, other input files (INCAR, POTCAR, KPOINTS, and LSF script) are automatically generated. Then, the subsequent NEB calculation can be performed by running the Load Sharing Facility (LSF) script.

### Data interaction: web interface

The web interface provides five functions: Materials Search, Materials Calculation, CIF Upload, Data Download, and Task Monitor. The operation flow of the web interface is elaborated as follows.

**Fig. 11:**
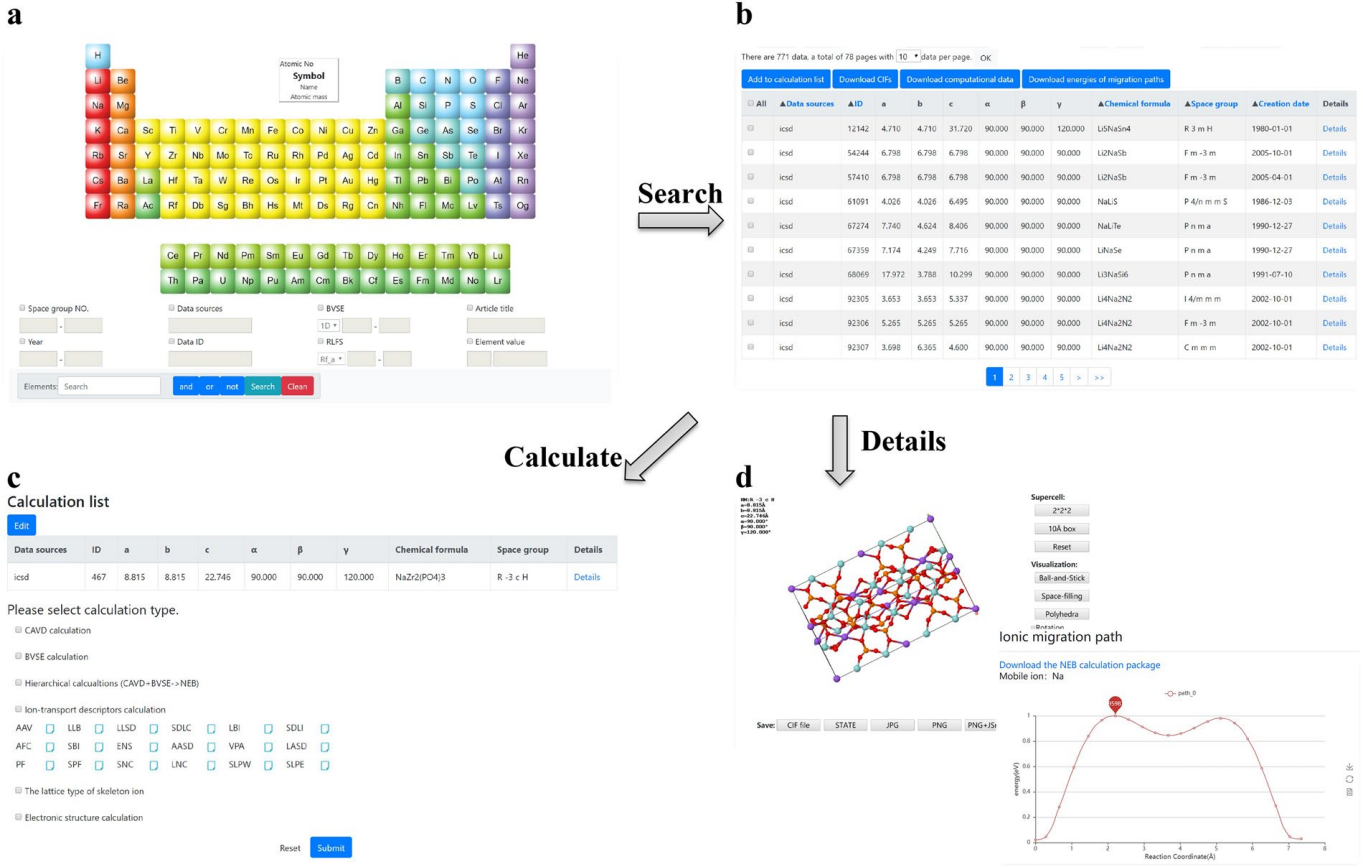
(**a**) Search page. (**b**) Results of a “Li & Na” search. (**c**) Materials calculation page. (**d**) Structure details page: structure visualization of NaZr_2_P_3_O_12_ and migration energy profile of Na ion in NaZr_2_P_3_O_12_.

First, the Materials Search page presents a periodic table and search options including the space group number, range of BVSE values, elements, and so on (Fig. 11a). The elements can also be directly selected from the periodic table. For instance, 771 compounds containing Li and Na are obtained by searching for “Li & Na” (Fig. 11b). The search results provide common information about the structure, including the data source, data identifier, lattice constant lengths, lattice constant angles, chemical formula, space group, and creation date. More details about the structure can be obtained by clicking “Details” to access the structure details page (Fig. 11d). Additionally, data can be downloaded in batches for analysis by clicking the “Download CIFs” or “Download computational data” button. In consideration of the demand for additional structures, a “CIF Upload” option is provided for users to upload CIF(s).

Second, the search page allows users to select the structure(s) of interest and click the “Add to calculation list” button to jump to the Materials Calculation page (Fig. 11c). Currently, CAVD, BVSE, ion-transport descriptor, and hierarchical (CAVD + BVSE→NEB) calculations are available. The parameter settings for these calculations are simple. For CAVD, the required parameters are the type of mobile ion and a threshold about the screening radii of bottlenecks and interstices. If the size of the interstice or bottleneck is within this threshold, it means that mobile ion can access the interstice or bottleneck. Similarly, the type and valence of the mobile ion are required for BVSE, and the grid resolution is an optional parameter with a default value of 0.1 Å. Grid resolution represents the distance between grid points. The lower the value is, the more accurate the calculation result will be. For hierarchical calculations, it not only involves the parameter setting of CAVD and BVSE, but also the screening values need to be set. If the radii of the largest free spheres calculated by CAVD and energy barriers calculated by BVSE are not within the range of the screening values, the hierarchical calculations will not continue. In addition, no parameters are required for the calculation of ion-transport descriptors.

Finally, the calculation tasks will be uploaded to our server after the calculation types are selected and the calculation tasks are submitted. Users can query the states of their submitted tasks using Task Monitor (the states include READY, RUNNING, COMPLETED, FIZZLED, etc.). The calculation results will be displayed on the structure details page for querying and downloading. For instance, the BVSE data file can be downloaded for visualizing the migration paths in VESTA^52^, and the NEB calculation package can be downloaded for external standalone VASP calculations.

### Machine learning: ion-transport descriptors

SPSE data can be used in machine-learning algorithms (such as linear regression, support vector machines, etc.) to predict materials properties and accelerate materials discovery and design^65^. For materials property prediction, the descriptors play an important role. Here, we provide 22 ion-transport descriptors, 20 of which are derived from the work of Sendek *et al*.^66^. The other two descriptors, RLFS and *E*_a_ values, are described as follows.

(1) RLFS: Radii of the largest free spheres calculated by CAVD

The largest free spheres calculated for the three principal directions (with corresponding radii *R*_a_, *R*_b_, and *R*_c_, respectively).

(2) *E*_a_ values: Energy barrier values calculated by BVSE

Approximate energy thresholds along the one-dimensional, two-dimensional, and three-dimensional migration paths.

The *E*_a_ values calculated by BVSE can be used as the decision attributes for activation energy prediction; the other 21 descriptors can be combined with regression analysis methods to predict the ionic conductivity. It is advantageous to accelerate the screening for solid electrolytes with high ionic conductivity and low activation energy, which are important preconditions of this screening^67–69^.

## Discussion

In this paper, we report the development of a high-throughput screening platform for solid electrolytes, SPSE. SPSE provides an open web interface for users to access a database and calculation tools of ion-transport properties, which are relatively lacking in the recent emerged platforms. Based on this, user can access the platform to complete the batch calculation and screening of the structures. The critical feature of SPSE is the fully automatic hierarchical calculations based on the analysis of crystal structure, and the implement of high-throughput calculation workflow. The hierarchical calculations combine empirical CAVD and BVSE calculations to obtain the ion-transport networks of crystal structures and then automatically analyzes these networks to obtain the approximate MEPs. These steps replace the linear interpolation method to provide more reliable migration paths for NEB calculation, avoiding unphysical paths and complicated manual preprocessing. This process enables high-throughput screening for potential solid electrolytes. It should be noted that the CAVD and hierarchical calculations are not applicable for crystal structure with fractional or mixed occupancy. The vision for further development of SPSE includes more sophisticated analysis of the hierarchical calculations, such as automatic molecular dynamics simulations, phase diagram calculation, etc. In addition, the ionic conductivity is helpful to screen the promising solid electrolyte. We intend to use the platform to obtain this information based on the BVSE calculation, and we have done the ionic conductivity prediction in our recent paper^50^.

## Methods

For CAVD calculation, the threshold about screening radii of bottlenecks and interstices needs to be set. The lower threshold is set to 0.563 Å for Li-containing compounds, and for Na-containing compounds it is 0.9 Å. Since the upper threshold is not considered in this paper, it is set to 3 Å. At present, we have provided a reliable reference range of the threshold in our resent paper^35^. In the BVSE calculation, the valence state of mobile ion is usually same with that in the CIF file, and the grid resolution is set as 0.1 Å.

For hierarchical calculations, the screening value of CAVD presents the range of RLFS, it can reference the threshold in the paper^35^. The screening value of BVSE is set between 0–1.2 eV in one-dimensional migration paths. The number of 1.2 is a suitable threshold to screen structures with low activation energy^51^. The NEB calculation is implemented in VASP and climbing image NEB method^70^ is selected by default. For all the VASP calculations, the exchange correlation of electrons is described by the Perdew–Burke–Ernzerhof (PBE) parameterization of the generalized gradient approximation (GGA)^71^. The plane-wave cut-off energy is set to 1.5 times larger than the maximum cut-off energy in POTCAR, and the k-point mesh is generated using the Monkhorst–Pack scheme^72^. The shape and volume of the unit cell are fixed at the optimized geometry. For the halting criteria for performing the NEB method and relaxing the end point structures, we provide a looser parameter for the convergence thresholds of the energy and force, which are set to 10^−4^ eV and 0.02 eV/Å, respectively. While the user can tune these parameters as their requirement. For the VASP calculations in the manuscript and supplementary information, the convergence thresholds are set as described above. All the preset settings in the VASP calculations have been tested.

## Supplementary information

Supplementary Information

## Data Availability

The authors declare that the main data supporting the finding of this study are available within the article and its Supplementary Information files. All the SPSE data have been deposited at figshare^73^.

## Code availability

The CAVD, BVSE and hierarchical computational codes have been integrated in SPSE, and they can only be run if the user has access to a SPSE account. The SPSE manuals are available in the website: https://www.bmaterials.cn/static/help/SPSE-UserManuals.pdf. Source codes of SPSE are freely available for download at figshare^73^.
