# Supplementary material for: High-throughput screening platform for solid electrolytes combining hierarchical ion-transport prediction algorithms
Source: Sci Data. 2020 May 21;7:151. doi: 10.1038/s41597-020-0474-y (PMC7242435; doi:10.1038/s41597-020-0474-y)
Supplement: Supplementary file 1 — Supplementary Information [file 41597_2020_474_MOESM1_ESM.docx]

High-throughput screening platform for solid electrolytes

combining hierarchical ion-transport prediction algorithms

Bing He^1^, Shuting Chi^1^, Anjiang Ye^1^, Penghui Mi^1^, Liwen Zhang^2^, Bowei Pu^2^, Zheyi Zou^2^, Yunbing Ran^2^,

Qian Zhao^3^, Da Wang^2^, Wenqing Zhang^4^, Jingtai Zhao^5^, Stefan Adams^6^, Maxim Avdeev^7,8^, Siqi Shi^2,3,*^

^1^*School of Computer Engineering and Science, Shanghai University, Shanghai 200444, China*

^2^*State Key Laboratory of Advanced Special Steel, School of Materials Science and Engineering,*

*Shanghai University, Shanghai 200444, China*

*^3^Materials Genome Institute, Shanghai University, Shanghai 200444, China*

^4^*Department of Physics and Shenzhen Institute for Quantum Science and Technology, Southern University of Science and Technology, Shenzhen, Guangdong 518055, China*

^5^*School of Materials Science and Engineering, Guilin University of Electronic Technology, Guilin 541004, China*

^6^*Department of Materials Science and Engineering, National University of Singapore, Singapore 117579, Singapore*

^7^*Australian Nuclear Science and Technology Organisation, Locked Bag 2001, Kirrawee DC NSW 2232, Australia*

^8^*School of Chemistry, The University of Sydney, Sydney 2006, Australia*

*corresponding author: Siqi Shi (sqshi@shu.edu.cn)

# **Preliminary screening of solid electrolytes**

In total, 5,192 Li- and Na-containing compounds from our materials database are identified as potential solid electrolyte candidates through preliminary screening for *E*_a_ $\leq$ 1.2 eV values in one-dimensional migration paths. The ion-transport properties of 32 solid electrolyte candidates are summarized in Table S1, including the radii of the largest free sphere in three directions produced by CAVD and the energy barrier values of three dimensional migration paths calculated using BVSE. Moreover, the GII (Global Instability Index)^1-2^ is a plausibility check for the crystal structure model. We can find that the GII of Li_2_O and Li_3_N are both greater than 0.2 which means the structural model of BVSE may not suitable for these structures. Among these 32 candidates, the disordered structure *β*-Li_3_PS_4_ with space group of *Pnma* (no. 62) has three different Wyckoff positions for lithium ions: Li1 (8d), Li2 (4b), and Li3 (4c). The Li1 site is fully occupied, and the Li2 and Li3 sites have occupancies of 70% and 30%, respectively. Here, we perform CAVD and BVSE calculations in *β*-Li_3_PS_4_-*b*, which has 100% occupancy of the *b* site.

**Table S1. Ion-transport properties of 32 solid electrolyte candidates. “RLFS” represents the radii of the largest free sphere calculated by CAVD. “Custom” in the identifier indicates that the structure is obtained from a custom CIF file. The Global Instability Index, i.e. here serves as a plausibility check for the crystal structure model.**

| No | Identifier | Compound | Space group | GII | RLFS (Å) | | | *E*_a_ by BVSE (eV) | | |
| --- | --- | --- | --- | --- | --- | --- | --- | --- | --- | --- |
|  |  |  |  |  | a | b | c | 1D | 2D | 3D |
| 1 | ICSD_201935 | LiZr_2_(PO_4_)_3_ | R-3c | 0.120 | 0.974 | 0.974 | 0.974 | 0.391 | 0.391 | 0.391 |
| 2 | ICSD_171375 | LiBF_4_ | P3_1_21 | 0.022 | 0.724 | 0.724 | 0.798 | 0.146 | 0.186 | 0.186 |
| 3 | ICSD_108886 | Li_2_O | R-3m | 0.481 | 0.723 | 0.723 | 0.723 | 0.127 | 0.127 | 0.127 |
| 4 | ICSD_173175 | Li_3_N | P6/mmm | 0.473 | 1.342 | 1.342 | 0.789 | 0.195 | 0.195 | 0.205 |
| 5 | ICSD_414242 | *β-*LiI | P6_3_mc | 0.016 | 0.537 | 0.537 | 0.546 | 0.254 | 0.254 | 0.254 |
| 6 | ICSD_054396 | Li_2_S | Fm-3m | 0.135 | 0.652 | 0.652 | 0.652 | 0.293 | 0.293 | 0.293 |
| 7 | ICSD_009082 | Li_5_GaO_4_ | Pbca | 0.142 | 0.691 | 0.691 | 0.688 | 0.440 | 0.440 | 0.469 |
| 8 | ICSD_035277 | LiAlCl_4_ | P2_1_/c | 0.044 | 0.578 | 0.553 | 0.529 | 0.332 | 0.332 | 0.352 |
| 9 | ICSD_062137 | Li_6_ZnO_4_ | P4_2_/nmc | 0.176 | 0.594 | 0.594 | 0.594 | 0.371 | 0.381 | 0.381 |
| 10 | ICSD_016229 | Li_5_AlO_4_ | Pmmn | 0.113 | 0.634 | 0.640 | 0.577 | 0.371 | 0.508 | 0.518 |
| 11 | ICSD_421083 | Li_6_PS_5_I | Cc | 0.157 | 0.517 | 0.517 | 0.513 | 0.332 | 0.342 | 0.361 |
| 12 | ICSD_002929 | LiAlSiO_4_ | P6_4_22 | 0.064 | 0.852 | 0.852 | 0.896 | 0.293 | 0.645 | 0.645 |
| 13 | ICSD_095979 | LiTi_2_(PO_4_)_3_ | R-3c | 0.106 | 0.757 | 0.757 | 0.757 | 0.752 | 0.752 | 0.752 |
| 14 | ICSD_157654 | Li_7_P_3_S_11_ | P-1 | 0.217 | 0.642 | 0.642 | 0.642 | 0.342 | 0.410 | 0.410 |
| 15 | ICSD_020208 | γ-Li_3_PO_4_ | Pnma | 0.043 | 0.611 | 0.621 | 0.611 | 0.479 | 0.479 | 0.820 |
| 16 | ICSD_073217 | Li_6_FeCl_8_ | Fm-3m | 0.027 | 0.461 | 0.461 | 0.461 | 0.488 | 0.488 | 0.488 |
| 17 | ICSD_246817 | Li_7_La_3_Zr_2_O_12_ | I4_1_/acd | 0.184 | 0.560 | 0.560 | 0.563 | 0.459 | 0.576 | 0.576 |
| 18 | ICSD_062244 | Li_3_Fe_2_(PO_4_)_3_ | P2_1_/c | 0.055 | 0.686 | 0.686 | 0.672 | 0.596 | 0.596 | 0.664 |
| 19 | ICSD_074950 | Li_7_TaO_6_ | P3 | 0.098 | 0.625 | 0.625 | 0.596 | 0.439 | 0.439 | 0.527 |
| 20 | Custom_400005 | *β*-Li_3_PS_4_-*b* | Pnma | 0.086 | 0.556 | 0.666 | 0.611 | 0.273 | 0.293 | 0.420 |
| 21 | ICSD_100403 | Li_2_GeO_3_ | Cmc2_1_ | 0.097 | 0.614 | 0.614 | 0.629 | 0.283 | 0.576 | 1.143 |
| 22 | ICSD_001180 | Li_8_SnO_6_ | R-3 | 0.165 | 0.588 | 0.588 | 0.583 | 0.557 | 0.557 | 0.645 |
| 23 | ICSD_065177 | Li_4_GeO_4_ | Cmcm | 0.081 | 0.626 | 0.605 | 0.741 | 0.527 | 0.703 | 0.703 |
| 24 | ICSD_008222 | Li_4_SiO_4_ | P2_1_/m | 0.086 | 0.592 | 0.650 | 0.582 | 0.557 | 0.791 | 0.801 |
| 25 | Custom_400004 | Li_10_GeP_2_S_12_ | P4_2_mc | 0.099 | 0.641 | 0.641 | 0.641 | 0.537 | 0.557 | 0.557 |
| 26 | Custom_300100 | Li_6_PO_5_Cl | F-43m | 0.196 | 0.391 | 0.391 | 0.391 | 0.645 | 0.645 | 0.645 |
| 27 | ICSD_019002 | Li_3_VO_4_ | Pmn2_1_ | 0.046 | 0.606 | 0.589 | 0.624 | 0.605 | 0.615 | 1.328 |
| 28 | ICSD_050420 | Li_3_Sc_2_(PO_4_)_3_ | P2_1_/c | 0.223 | 0.720 | 0.720 | 0.650 | 0.605 | 0.723 | 0.762 |
| 29 | ICSD_165579 | LiClO_4_ | Pnma | 0.028 | 0.616 | 0.677 | 0.616 | 1.162 | 1.465 | 1.621 |
| 30 | ICSD_020740 | Na_3_Sc_2_(PO_4_)_3_ | R-3c | 0.041 | 0.992 | 0.992 | 0.991 | 0.977 | 0.977 | 0.986 |
| 31 | ICSD_000467 | NaZr_2_(PO_4_)_3_ | R-3c | 0.048 | 1.001 | 1.000 | 1.000 | 1.045 | 1.045 | 1.055 |
| 32 | ICSD_015970 | Na_2_O(Al_2_O_3_)_11_ | P6_3_/mmc | 0.164 | 1.271 | 1.271 | 0.326 | 0.117 | 0.117 | - |

# **Non-equivalent migration paths of Li_7_La_3_Zr_2_O_12_**


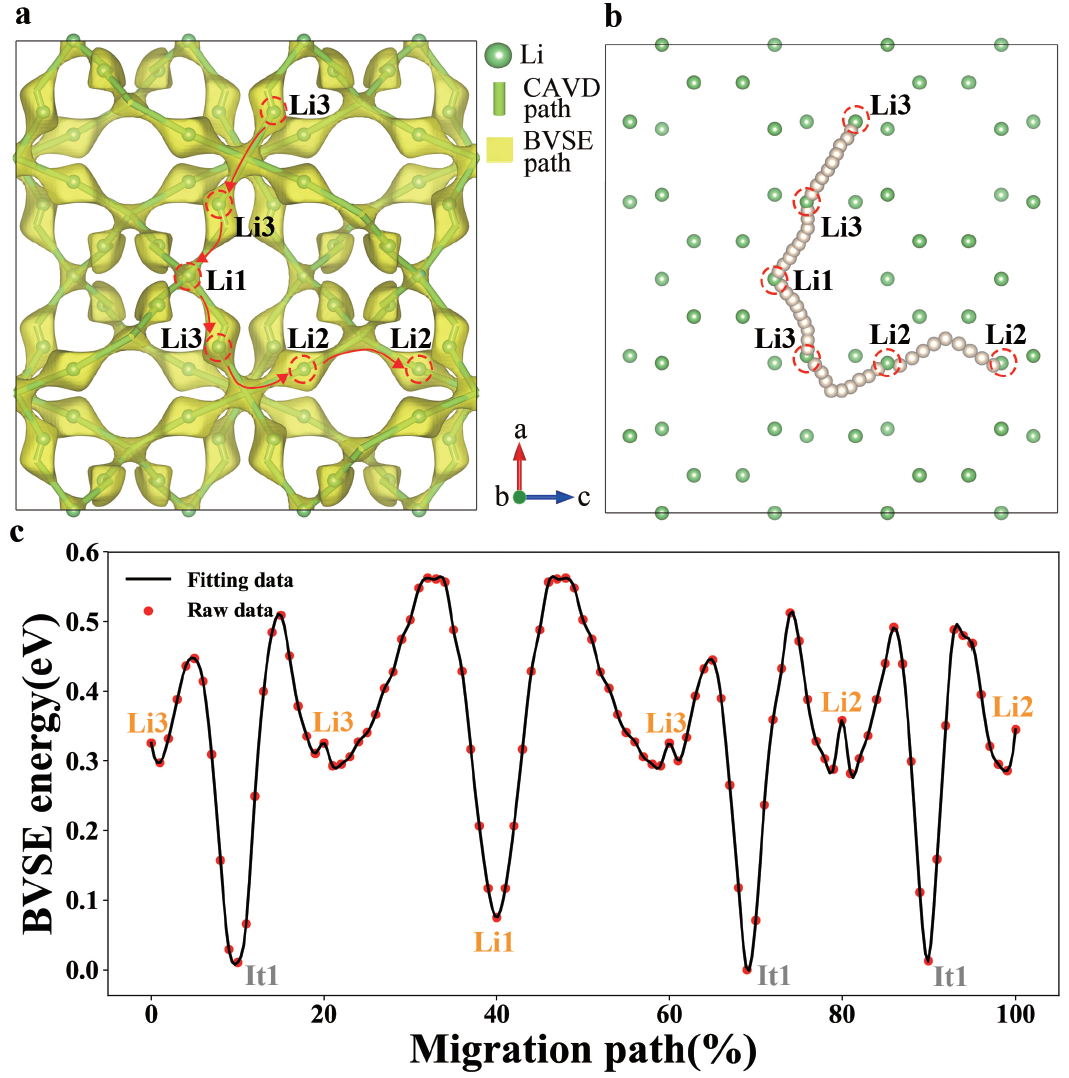


**Figure S1.** The ion migration paths of **a** LLZO calculated using CAVD and BVSE are shown in green cylinders and yellow isosurfaces, respectively. The endpoints of migration paths are highlighted by the red dotted circles. **b** Approximate MEPs and **c** migration energy profiles of Li1–Li3, Li2–Li3, Li2–Li2, and Li3–Li3 in LLZO; Li3-Li1 is equivalent to Li1-Li3.

In tetragonal Li_7_La_3_Zr_2_O_12_, *E*_a_ of the three-dimensional migration paths is 0.576 eV and the threshold parameter related to the radius of Li^+^ is 0.563 Å (Figure S1a). There are four types of non-equivalent migration paths: Li1–Li3, Li2–Li2, Li2–Li3, and Li3–Li3. Among them, Li2–Li2, Li2–Li3, and Li3–Li3 all pass through the same interstitial site it1. The MEPs and energy profiles are shown in Figure S1b and Figure S1c, respectively.


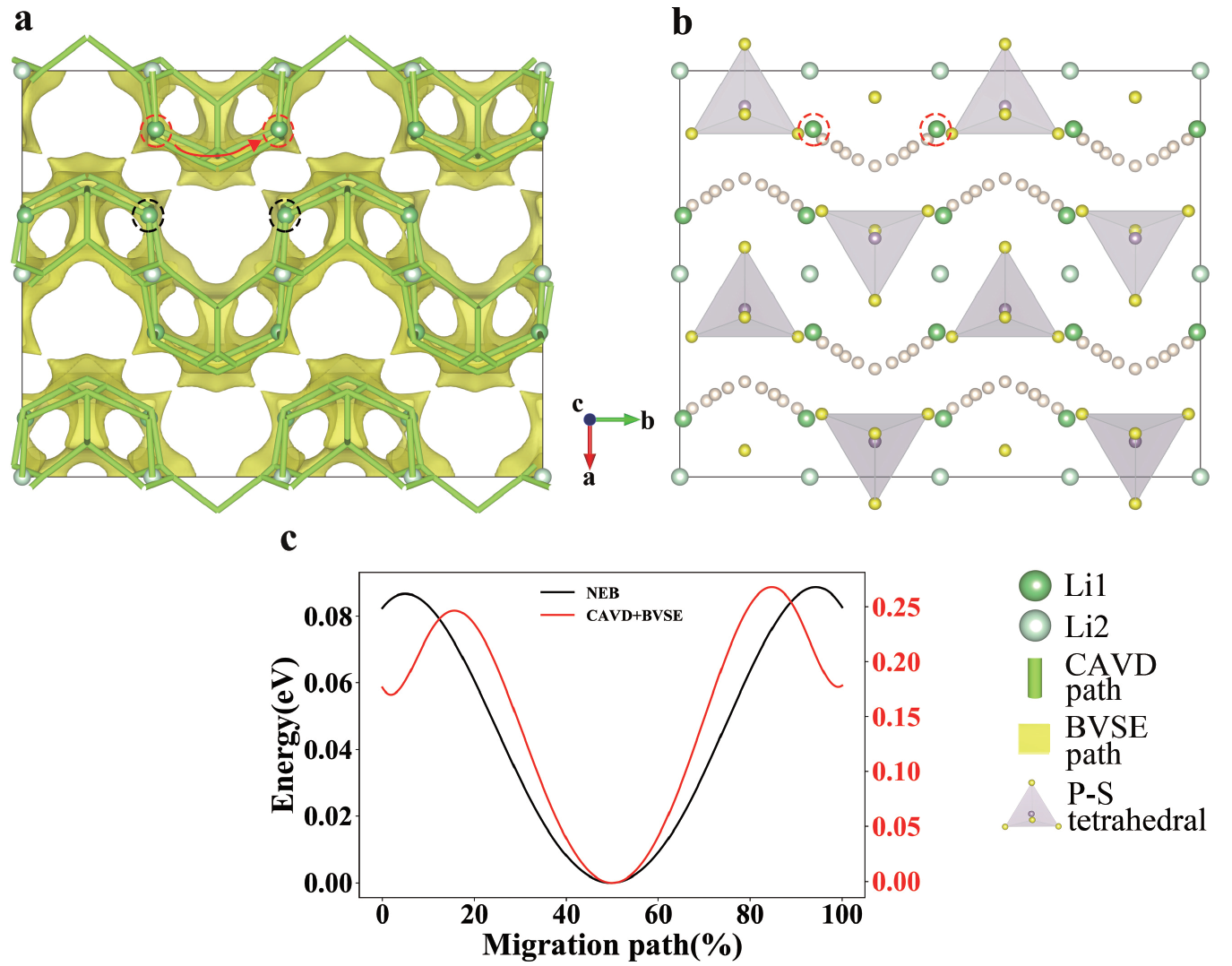


**Figure S2.** **a** The green cylinders represents the ion-transport network of *β*-Li_3_PS_4_-*b* calculated using CAVD with the threshold parameter related to radius of Li^+^ to be 0.563 Å. The yellow isosurfaces of *β*-Li_3_PS_4_-*b* are calculated using BVSE with *E*_a_ of 0.42 eV. The endpoints of the path are highlighted by red dotted circles. **b** An approximate MEP of endpoints generates other equivalent paths in the super cell. **c** Energy profiles are calculated using FP-NEB and CAVD + BVSE methods, respectively.

# **Equivalent paths of *β*-Li_3_PS_4_-*b***

The approximate MEP of Li1–Li1 in *β*-Li_3_PS_4_-*b* calculated by hierarchical calculation is shown in Figure S2. The path passes through two bottlenecks and one interstice. We created two vacancies along the path to eliminate the Coulomb repulsion between Li ions in a 1×2×2 super cell (the two vacancies are highlighted by the black dotted circles in Figure S2a). In this case, the energy profile calculated using the FP-NEB method is virtually consistent with that calculated using the BVSE + CAVD method (Figure S2c).

# **References**

1. Thangadurai, V., Adams, S. & Weppner, W. Crystal Structure Revision and Identification of Li^+^ -Ion Migration Pathways in the Garnet-like Li_5_La_3_M_2_O_12_(M = Nb, Ta) Oxides. *Chem. Mater*. **16**, 2998–3006 (2004).
2. Salinas-Sanchez, A., Garcia-Muñoz, J., Rodriguez-Carvajal, J., Saez-Puche, R. & Martinez, J. Structural characterization of R_2_BaCuO_5_ (R = Y, Lu, Yb, Tm, Er, Ho, Dy, Gd, Eu and Sm) oxides by X-ray and neutron diffraction. *J. Solid State Chem*. **100**, 201–211 (1992).
